# Views and Treatment of an Important Injury of the Wrist

**Published:** 1838-11-07

**Authors:** J. Rhea Barton


### *Views and Treatment of an important Injury of the Wrist.* By J. Rhea Barton, M. D.

Any further observations on a class of accidents, so common, and which have been so often the subject of inquiry, as that of injuries of the forearm and wrist-joints, may be deemed superfluous by those who read, but have no personal experience in surgery. But to those engaged in the active pursuits of our profession, it is well known that, notwithstanding the volumes that have been written on this subject, there are yet certain injuries involving these parts which are not fully understood, and consequently not successfully treated.

My attention was early fixed upon such cases, and through a series of years they have been particularly interesting to me; and it is from my conviction that, up to this time, error prevails, both as to the nature and the treatment of them, that I am induced to publish my views and practice therein.

I do not know any subject on which I have been more frequently consulted than on deformities, rigid joints, inflexible fingers, loss of the pronating and supinating motions, and on neuralgic complaints resulting from injuries of the wrist, and of the carpal extremity of the forearm—one or more of these evils having been left, not merely as a temporary inconvenience, but as a permanent consequence.

The accidents which are to be the principal subject of my remarks, usually pass either for sprains or dislocations of the wrist. Under one of these denominations are these cases to be detected, which, though partaking somewhat of the character of sprains or dislocations, are distinguishable from either of them respectively. They may be recognised by their being accompanied by more distortion of the hand and arm than any which can arise from simple sprains of the wrist, and yet less than that which must necessarily take place when there exists a complete luxation of the carpus. The profile of the limb under this injury is a peculiar one, distinguishing it on the one hand from the sprained wrist, and on the other from luxation.

A nice discrimination between these and the other varieties of accidents is not a mere matter of useless refinement in diagnosis; but it is one of great practical importance, as is confirmed by the number of persons who have never fully recovered from the effects of accidents of this nature, treated without such discrimination.

In simple sprains of the wrist, though accompanied by extreme swelling, the limb will still be found to retain a characteristic outline of its natural contour. It is not marked by any abrupt and solid eminences, the swelling is rather uniform, diffuse, and puffy, the hand continues on the same line with that of the forearm, &c. In complete dislocations, the nature of the injury must always be very palpable from the great bulging of the overlapped bones, and from the shortening of the limb, &c. Between these two injuries there is too great a dissimilarity to admit of an excuse for the surgeon who mistakes the one for the other; but he may confound with these, and it is a common fault to do so, *a sub-luxation of the wrist, consequent to a fracture through the articular surface of the carpal extremity of the radius;* although to this accident belong appearances exclusively its own.

It is to this peculiar injury that I wish to draw attention.

It is one of the most common injuries to which the upper extremities are subjected; and every practitioner of moderate experience will, I am sure, be able to call to his recollection the appearance which the limb presents under such circumstances, as well as the embarrassment which he has experienced in his attempts to obviate eventual deformity, to preserve the functions of the fingers, and to restore the motions of the wrist and forearm.

The similarity of manner in which this accident generally occurrs, is striking. It is almost always found to have taken place in consequence of the individual having thrown out his hand to rescue himself from falling, or to ward off injuries threatening a more important part of the body. In the act of falling, for example, the hand is thus instinctively thrown out, and the force of the fall is first met by the palm of the hand, which is violently bent backward until the bones of the wrist are driven against the dorsal edge of the articulating surface of the radius, which, being unable to resist, it gives way. A fragment is thus broken off from the margin of the articular surface of this bone, and is carried up, before the carpal bones, and rested upon the dorsal side of the radius; they having been forced from their position, either by the violence, or by the contraction of the muscles alone. We have then an imperfect luxation of the wrist, depending on a fracture through the extremity of the radius. The deformity will be found to correspond with this state of the case. There is a tumour on the dorsal side of the arm formed by the bulging of the carpal bones and fragments; whilst below it, on the palmar side, the extremity of the radius projects. The degree of prominence of these parts, depends upon the size of the fragment and the violence of the injuring force. The ulna not being very intimately involved in the injury, retains its position, and serves as an abutment, against which the hand seems to rest; whilst the radius, as it has its edge broken off, allows the hand on that side to be drawn upward, and hence to render, on the under side, the styloid process of the ulna more conspicuous than natural. Crepitus cannot always be felt, sometimes in consequence of the smallness or crushed condition of the fragment; at other times, owing to the great swelling and tension; but in every such case, the distortions of the limb are to be seen, and may be removed by making firm extension and counter-extension from the hand and elbow, at the same time gently depressing the tumours already spoken of. By the employment of these means, all deformity, except that which evidently depends upon the more general swelling, may be satisfactorily removed; but the moment the extension and counter-extension are relaxed, the combined action of the flexors and extensors of the fingers, as well as those of the wrist, force the deformity to re-appear as conspicuously as before: and as often as the effort is renewed and discontinued, will the deformity appear and disappear. In this respect does this species of injury in an especial manner differ from a complete simple luxation of the wrist; which, when once reduced, must continue so after the reducing force has been withdrawn. There is no spontaneous reluxation after the simple complete dislocation has been removed; whereas, in this case it immediately succeeds the withdrawal of the force. This accident must not be confounded with those which are also of frequent occurrence, namely, fracture of the radius, or of the radius and ulna just above, and not involving the joint. It will be found on referring to the writings of Boyer, Desault, Sir Astley Cooper, Dupuytren, and many others, that this frequently happens, and that the fracture often reaches to within a few lines of the extremity of the bone; and that these cases are very frequently mistaken for dislocations, though they are in reality fractures exterior to and disconnected with the joint; the deceptive deformity being occasioned by the displacement of the broken ends of the bone caused by the action of the muscles and the weight of the hand. A very good illustration of such cases may be found in plate 12, figure 1, in Mr. Hind's folio work on fractures of the extremities. It may there be seen how powerfully the flexors and extensors act in retracting the inferior portions of the bones, and how closely the radius and ulna are drawn together through the instrumentality of the pronator quadratus muscle below, whilst toward the brachius the pronator teres is exerting its power to keep the limb in a state of pronation. Now these are consequences which do not result from the species of injury to which I refer. The fragment may be, and usually is, quite small, and is broken from the end of the radius on the dorsal side, and through the cartilaginous face of it, and necessarily into the joint. The pronator quadratus is not involved in the fracture. The radius and ulna are not materially disturbed in their relations to each other. The only important change, which takes place in consequence of this fracture is, that the concave surface at the extremity of the radius, which receives and articulates with the three first carpal bones, is converted, as it were, into an oblique surface by the loss of a portion of its marginal ridge; commonly by the separation of an entire piece; sometimes by the crushing of its substance. The moment the cartilaginous extremity of the radius is deprived of its concave form, the united force of the carpal and digital flexors and extensors is exerted to create a complete luxation; but as the ligaments are only stretched, or but partially torn, this cannot take place. The carpal bones, therefore, only emerge collectively from their natural position, and carrying before them the broken piece, rest on the dorsal side of the radius, forming a tumour there; whilst the end of the radius itself occasions on the palmar side a prominence which is round and smooth, and differing in this from similar projections formed by the fractured ends of bones, the abruptness and harshness of which may sometimes be distinctly felt through the soft parts, and which are themselves, when pressed upon, acutely painful.

It follows of course, in injuries of this kind, that unless some method of dressing be adopted whereby the retraction of the hand may be permanently counteracted, and the prominences repressed, the patient will recover with a crooked arm, and under a sacrifice of some of the functions of the hand. The customary modes of treating either sprains or dislocations of the wrist, or fracture of the forearm, are totally inadequate to the purpose, and should not be relied on as a treatment for these particular cases by any practitioner who has regard for the welfare of his patient, and for his own reputation. There is no professional point upon which I can more confidently express myself, than upon the errors committed in the treatment of these cases,—passing, as they commonly do, for sprains of the wrist, and hence treated as such. After an unvarying success in the management of this accident for many years in the Pennsylvania Hospital, in the Blockley Hospital, and in private practice, I can strongly recommend the following plan of treatment:—Two thin, but firm splints of wood, are to be prepared, of sufficient length to extend from just below the condyles of the os humeri to the ends of the fingers, and of width enough to embrace the sides of the limb. These are to be lined on one of the sides with carded cotton, or something equally soft, and wrapped with a bandage. Two compresses, each about two inches square, and composed of strips of bandage, about one yard and a half long, evenly folded up, are also to be in readiness. The arm is then to be flexed at the elbow, and one assistant is to hold it firmly above the condyles, whilst another makes extension from the fingers. The surgeon now presses the prominent end of the radius on the inner side, and the bulging carpus and fragment on the outer side, into their respective places. The roller is then to be lightly pressed around the hand and arm, securing in its course up the limb one of the compresses precisely over the carpus and back of the hand, and the other with equal precision over the palmar side of the radius just above its carpal extremity. These compresses, when properly arranged, will be found *not opposite to each other, but the inner one commencing on a line opposite to that on which the outer one has terminated.* These being applied, the inner splint is next placed against the limb,—the assistant shifting his hand to admit of this being done, without his relaxing in the least degree the extension until the limb is bandaged to this splint, when it will be found that the extension is well maintained. The outer splint is now to be applied and secured to the arm by the return of the roller. The principal use of the latter splint is to act upon the outer compress, and by its general pressure to weaken for the time the force of the resisting muscles. By the employment of these simple means, the indications in the treatment of this accident will be found to be fully met. The arm may be carried in a sling, and the patient permitted to walk about, &c. In three or four days the limb should be undressed and inspected; and whilst held so that relaxation cannot take place, the wrist and fingers are to be bent enough to preserve the flexibility of the joints. The dressings are then to be reapplied. These operations are thenceforward, for four or five weeks, to be repeated every day, adding to them the motions of pronation and supination.

The practice of keeping a limb in splints, with the joints in an immoveable state for weeks, even when the fracture is remote from the articulation, cannot be too earnestly deprecated; and in cases where the injury to be repaired has involved a joint, such treatment is censurable to a high degree, as it is almost certain to destroy the mobility of it by promoting the adhesion of ligaments, the union of tendons with their thecæ, and by obliterating bursæ—evils never to be fully repaired. So prevalent is the error on this point, and so serious are the results of such practice, that I have settled my mind to the belief, that in very many cases of fractures the imperfect recovery of the patient is owing to the injudicious use of splints and bandages, rather than to the complication or original difficulty of the case. For the interruption of adhesions of the ligaments, for insuring a continuance of the muscular power and offices of the tendons, and for the entire preservation of the motions of joints, it is indispensably necessary that these parts should be put into action frequently during the treatment of a fracture in which they are interested, either from the adjacency of the fracture, or from their confinement by the splints necessarily used on the occasion. The movement of these parts by the surgeon at stated periods, is not at all incompatible with the quietude and the progressive reunion of the bone itself. The omission of this duty arises, I am persuaded, out of our knowledge of the necessity of securing rest to a broken bone, without at the same time considering that by the means we employ, and the course we pursue to accomplish it, we may entail upon our patient a calamity quite as deplorable as that of an ununited fracture or a crooked bone—namely, a stiff and useless limb. The surgeon, then, is to recollect, that in the cases made herein the special subject of notice, he has not only the duty to perform of obviating deformity of the limb, but of preserving the free motions of all the other parts, and that this can be accomplished only by daily trials of their freedom and functions.

By an adherence to the plan of treatment just recommended, and by an attentive pursuance of the means spoken of to preserve the functions of the limb, I have uniformly succeeded in restoring perfectly the arm to its natural shape and offices. I can, consequently, on just grounds, advise others to adopt the same practice.

It sometimes happens, also, though rarely, that fracture of a similar character to the one just described, occurs *on the palmar side* of the radius, from the application of force against the back of the hand whilst it is bent forward to its ultimate degree. This usually happens in awkward attempts to parry the blow of a fist, from pressure in dense crowds, and from falling on the back of the hand whilst it is bent forward. Whenever the fracture takes place in front, the end of the radius projects over the wrist on the dorsal side, and the carpal bones and fragment rise out of their proper situations, and form the tumour on the palmar side, thus reversing the deformity of the arm. The principle in the treatment of this variety of the injury, is the same as in the foregoing.

Dupuytren used to trace an analogy between the ordinary fracture of the lower end of the radius, and fracture of the lower end of the fibula; and as he had founded a very successful method of treating the latter injury from the view he took of such cases, he extended his analogy to the treatment of the former by means and apparatus designed to accomplish the same ends. How far the practice may be successful when applied to the cases for which the practice was specially intended, I cannot say. Having myself found simpler means attended with success, I never adopted his practice; but for the treatment of fracture through the joint, &c. the practice would be unavailing. Neither is there any resemblance of this injury to the fracture of the fibula. It may be, however, not inaptly compared to the partial luxation of the foot, depending on fracture of the internal malleolar process of the tibia, including a portion of the articular face of the bone—an accident well known to surgeons.

